# An integrative method to normalize RNA-Seq data

**DOI:** 10.1186/1471-2105-15-188

**Published:** 2014-06-14

**Authors:** Cyril Filloux, Meersseman Cédric, Philippe Romain, Forestier Lionel, Klopp Christophe, Rocha Dominique, Maftah Abderrahman, Petit Daniel

**Affiliations:** 1 INRA, UMR1061, Unité Génétique Moléculaire Animale, 123 avenue Albert Thomas, F-87060 Limoges Cedex, France; 2 Université de Limoges, UMR1061, Unité Génétique Moléculaire Animale, 123 avenue Albert Thomas, F-87060 Limoges Cedex, France; 3 INRA, UMR1313, Unité Génétique Animale et Biologie Intégrative, Domaine de Vilvert, F-78352 Jouy-en-Josas, France; 4 AgroParisTech, UMR1313, Unité Génétique Animale et Biologie Intégrative, Domaine de Vilvert, F-78352 Jouy-en-Josas, France; 5 INRA, Sigenae, Chemin de Borde-Rouge, Auzeville, BP 52627 31326 Castanet-Tolosan Cedex, France

**Keywords:** Gene expression, Normalization, RNA-sequencing, qRT-PCR

## Abstract

**Background:**

Transcriptome sequencing is a powerful tool for measuring gene expression, but as well as some other technologies, various artifacts and biases affect the quantification. In order to correct some of them, several normalization approaches have emerged, differing both in the statistical strategy employed and in the type of corrected biases. However, there is no clear standard normalization method.

**Results:**

We present a novel methodology to normalize RNA-Seq data, taking into account transcript size, GC content, and sequencing depth, which are the major quantification-related biases. In this study, we found that transcripts shorter than 600 bp have an underestimated expression level, while longer transcripts are even more overestimated that they are long. Second, it was well known that the higher the GC content (>50%), the more the transcripts are underestimated. Third, we demonstrated that the sequencing depth impacts the size bias and proposed a correction allowing the comparison of expression levels among many samples. The efficiency of our approach was then tested by comparing the correlation between normalized RNA-Seq data and qRT-PCR expression measurements. All the steps are automated in a program written in Perl and available on request.

**Conclusions:**

The methodology presented in this article identifies and corrects different biases that influence RNA-Seq quantification, and provides more accurate estimations of gene expression levels. This method can be applied to compare expression quantifications from many samples, but preferentially from the same tissue. In order to compare samples from different tissue, a calibration using several reference genes will be required.

## Background

The study of transcriptome has pushed forward by the development of next-generation sequencing technologies. RNA-Seq offers the possibility to get information on sequence and quantification of all transcribed genes, but extremely lowly expressed ones [1]. As shown by these authors, this method differs from the microarrays which have limitations due to (*i*) the difficulty to design specific probes, leading to artifacts caused by cross-hybridization and (*ii*) the impossibility to detect expression for non-annotated genes. Expression quantification performed using qRT-PCR is more precise than microarrays, but is also not able to measure unknown genes. Moreover, the cost of TLDA (TaqMan Low Density Array - *Applied Biosystems*) for example, renders it unsuitable for large gene sets.

The RNA-Seq protocol is a succession of technical steps followed by quantification. According to *Illumina* technology, (*i*) a cDNA library from a given tissue is randomly fragmented by sonication, (*ii*) specific adapters are ligated for the assignation of each fragment to the corresponding sample, (*iii*) PCR amplification are performed, and (*iv*) amplified mRNA fragments with sizes ranging from 250 to 450 bp are isolated before being sequenced. The quantification of the sequenced fragments (called reads) begins with the mapping of each read onto the assembled genome or transcriptome, in order to count the number of reads assigned to each known or unknown gene. When there are several transcripts or close paralogues for a gene, the attribution of a read to the right transcript is not always possible depending on the read position: 5’-end fragments are expected to be more specific than 3’-end ones. The second step of quantification consists in removing four biases affecting read counts: (*i*) the number of reads increases with the size of the transcript [2-6], (*ii*) with the amount of the cDNA library [7,8], (*iii*) sequencing efficiency decreases when the GC-content is too low or too high [9-12], and (*iv*) due to a PCR amplification step during the library preparation, PCR duplicates occur when two copies of the same cDNA fragment produce different clusters on the flow cell [13-15].

Since RNA-Seq emergence, a number of normalization methods have been developed to address one or two of the different biases [1-12,14]. Our aim was to develop an integrated method able to correct all these sources of bias. In order to avoid RNA-Seq quantification problems linked to specific isoforms, unlike most studies, we only retained genes with a single transcript to determine the various equations and to perform the comparison [16]. As for size effects, most of them are based on mathematical distribution models to compare expression levels between samples, but do not consider separately the opposite biases relative to size: short transcripts (<600 bp) are underestimated while longer ones are overestimated. As for the bias linked to GC content, we performed simple regression methods based on polynomial model. It appeared that sequencing depth has an effect on the equations driving the size and GC content corrections. Hence, unlike other methods, a further run of our program was performed to correct globally the read counts by taking into account size, GC content and total read numbers. In order to assess the efficiency of our approach, we calculated the correlation between corrected RNA-Seq counts and qRT-PCR quantifications.

## Methods

### RNA extraction

*Longissimus thoracis* muscle biopsies were taken between the 7^th^ and 9^th^ ribs of 125 limousine bulls slaughtered at the age of 15.8 months. The samples were immediately frozen in liquid nitrogen and stored at -80°C. After grinding tissues using a FastPrep FP120 Homogenizer device (*Thermo Savant*) and micro-tubes “Lysing Matrix D” (*MP Biomedicals*), RNA extractions were performed with the RNeasy Midi/Maxi kit (*Qiagen*). The procedure and solution quantity were optimized for extraction from skeletal muscles and treatment with proteinase K as recommended by the supplier. The quality control of RNA step was done using RNA 6000 Nano Chips analyzed with 2100 Bioanalyzer instrument (*Agilent Technologies*). The 22 best ranking RNA samples were retained.

### RNA sequencing

To verify the absence of degradation during the storage period, the quality of these 22 cattle samples were then checked again before preparing cDNA libraries according to the *Illumina* protocol. Briefly, mRNAs were isolated from total RNA by their polyA tails and cDNA libraries were built using random-hexamers. These cDNAs were fragmented by sonication, and specific adapters were then ligated to each fragment for the traceability of the sample. Ten cycles of PCR amplification were performed. Amplified mRNA with a size between 250 and 450 bp were then isolated before being sequenced in paired-end reads with a length of 100 bases using *Illumina* HighSeq2000 device (hosted at the INRA Genomic Platform of Toulouse, France).

### RNA-Seq read counting

The first step consists in de-multiplexing the reads by recognizing specific adapter sequences to assign each read to the corresponding sample (three samples were pooled per flow cell lane). From 100 to 240 million paired-end reads were obtained per flow cell lane, corresponding to 27 to 91 million reads for each cDNA library. These paired-end reads were then mapped back to the bovine reference transcriptome, using *Bos taurus* known transcripts recorded in the Ensembl database v.61 (Website: ftp://ftp.ensembl.org/pub/release-75/fasta/bos_taurus/cdna/). This set contains 27,663 transcript sequences assigned to 21,734 known genes and pseudogenes. Paired-end reads located exactly on the same transcript were selected and counted. A total of 21,455 transcripts (17,605 genes) were identified, with at least one paired-end read within the 22 analyzed samples.

### qRT-PCR quantification

Among the 22 cattle samples, five of them were chosen to perform qRT-PCRs on the basis of a large range of total read numbers. These samples showed around 10.10^6^ (1475), 13.10^6^ (1455), 20.10^6^ (1479), 24.10^6^ (1345), and 30.10^6^ reads (1476), respectively. These experiments were conducted using custom-made TLDA (Taq-Man Low Density Array) cards and ABI PRISM 7900HT sequence detector system (*Applied Biosystems*). The dataset was built with genes involved in glycosylation metabolism, named glyco-genes in the following. They concern glycosyl-transferases, glycosidases, sulfo-transferases, sugar carriers, and lectines. Among the around 800 genes recorded in the bovine genome (unpublished data), 372 were selected according to two criteria: the greatest diversity of the glyco-gene groups and the availability of primers provided by *Applied Biosystems* (https://bioinfo.appliedbiosystems.com/genome-database/gene-expression.html). Twelve housekeeping genes (18S RNA, TFIID transcription factor, etc., see [17]) were added as controls to complete the 384-microwells of each microfluidic card. The quantification was done using the SDS 2.3 software (*Applied Biosystems*) according to the ΔCt method (see the *User Bulletin #2* for ABI PRISM 7700 of October 2001). Briefly, ΔCt corresponds to the threshold cycle (Ct) for each gene minus that of the mean of the twelve endogenous internal controls.

### RNA-Seq data from public datasets (drosophila and human)

To validate the first steps of our method, it was necessary to consider public data dealing with other organisms than *Bos taurus*.

As for Drosophila, in the public dataset SRA: SRP009459/GEO: GSE33905 deposited by B.R. Graveley and co-workers, we downloaded 16 read sequence sets obtained from head of male and female adults (GSM838758 to GSM838760, GSM838763 to GSM838766 to GSM838780, and GSM838799 to GSM838802). The sequencing depth varied from 2.7 to 8.4 million reads, with a mean value close to 5 million reads.

As for Human, we considered the dataset SRA: SRP032775/GEO: GSE52166 deposited by R. Sanka and co-workers. In order to have homogeneous data, the read sequence sets came from whole blood of 20 individuals in a pre-infection state relatively to *Plasmodium falciparum* (GSM1335718, GSM1335720, GSM1335722 to GSM1335756). The sequencing depth varied from 21.2 to 72.9 million reads, with a mean value around 40 million reads.

Using STAR aligner software v.2.3.1f [18], the read sequences were splice-aligned onto the Drosophila v.BDGP5.75 or the Human genome v.GRCh37.75, respectively. Transcripts were quantified with sigcufflinks (available upon request at http://www.sigenae.org), a modified version of the cufflinks code [19] providing raw read counts per transcript, by using the GTF reference files provided by Ensembl (version 75).

### Simulation of RNA-Seq data

As we suspected that the RNA fragment sizes have an impact on the behavior of read counts as a function of transcript sizes, it was useful to conduct simulation using a specific program. We first downloaded the transcript genes of *Bos taurus* chromosome 20 from Ensembl (version 75 – genome assembly UMD3.1). All the sequences were concatenated to obtain a single sequence of 255,601 bp. This sequence was then split into 231 genes in the FASTA format, with increasing sizes from 50 bp to 1,200 bp according to an arithmetic progression with common difference of 5. This file was submitted to rlsim [20]. Default parameters were chosen, except for sequenced fragment range, and total read number (1 million). Three runs were launched, the first with 250–450 (mean 350) bp, the second with 450–650 (mean 550) bp, and the last 650–850 (mean 750) bp. For each transcript, the program assigns an expression level from a mixture of gamma distribution with two components with mean 5,000 and 10,000. Then, the simulation provides for each read its sequence and the assigned gene. We then calculated the number of reads for each gene using the program Fishing-net, written in Perl, available upon request from CF and DP.

## Results

As qRT-PCR quantification were used to validate our RNA-Seq normalization method, it was necessary to verify that qRT-PCR data were not subject to transcript size and GC content biases. As for transcript size, we tested a relationship with the ΔCt obtained by qRT-PCR for cattle sample 1479 (n = 233). Through polynomial equations of first and third orders, the *p*-values were 0.84 and 0.87, respectively. As for GC content in the same sample, the corresponding polynomial equations gave *p*-values of 0.57 and 0.96, respectively. We verified that for the four other cattle samples, no significant relationships were observed neither for transcript size nor for GC content (Additional file 1).

To compare qRT-PCR results with the RNA-Seq approach, several steps of correction are needed. The calculations concerned 14,676 genes for which only one transcript were detected in Cattle. We propose an integrated method called SGTR (*transcript Size, GC content and Total Read number*) that takes into account the effects of transcript size, GC content, and total read number. First, it was necessary to apply a *log*_*2*_ transformation to raw counts to avoid large dispersion for high values according to [2,10,21] and [22].

### Correction of transcript length biases

**Figure 1:**
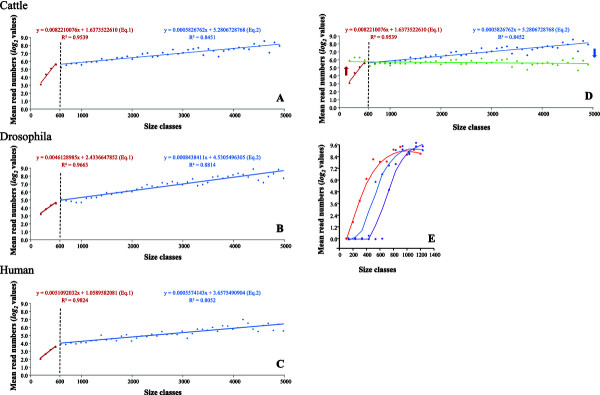
**Method implemented to correct the biases linked to transcript size.** Size classes were built every 100 bp for transcripts < 5000 bp, as too few transcript numbers were observed with a size ≥ 5000 bp leading to scattered dots. The dotted line separates the red regression line corresponding to transcripts < 600 bp and the blue one to transcripts ≥ 600 bp. The vertical axis corresponds to *log*_*2*_ transformed values of read numbers. **A)** Cattle sample 1479. **B)** Drosophila sample SRR384925 (7132 genes with a single transcript). **C)** Human sample SRR1177729 (16,228 genes with a single transcript). **D)** Representation of sample 1479 after correction (green). **E)** RNA-Seq simulated data using rlsim, where Loess smoothing was applied to each series. The blue points correspond to a run where the sequenced fragments are in the range of 250–450 bp, the red one to the range of 450–650 bp, and the violet one to the range of 650–850 bp.

We sorted the transcripts according to their size and built length classes: the class *n* contains all the transcripts for which the size is comprised in the [ *n* ; *n* + 99 ] interval. As for example, the cattle sample 1479 resulted in the Figure 1A. It is clear that two parts can be observed on both sides of the size 600. The regression equations for transcripts < 600 bp and ≥ 600 bp are respectively as follows:

(1) $$y_{i}=a_{1}.x_{i}+b_{1}$$

and

(2) $$y_{i}=a_{2}.x_{i}+b_{2}$$

where ***y***_***i***_ corresponds to the mean read number for the size class ***x***_***i***_.

We observed that the slope ***a***_***1***_ for shorter transcripts was higher than the one ***a***_**2**_ for longer transcripts, and verified that this trend was also true for the 21 other cattle samples analyzed by RNA-Seq. In particular, the 600 bp border remained constant. In other species (e.g. Drosophila and Human), we retrieved this 600 bp border in all the samples tested (16 drosophila head samples and 20 human whole blood samples). One example of each of this species is presented in Figures 1B and 1C. To correct the bias linked to transcript sizes, it was necessary to introduce two different equations corresponding to each part of the graph. As size 600 is a critical value, we decided to adjust all the read numbers to this size. First consider the left part; for a transcript of size ***S***, we added the value “ ***a***_***1***_**(600 -*S*)** ” to the observed read number. Likewise, for transcripts > 600 bp, we removed the value “ ***a***_***2***_**(*S*- 600)** ”. As a result, the read numbers of all the transcripts were adjusted to the size 600 (Figure 1D).To understand the significance of this 600 bp border, we hypothesized that it could be due to the length of the sequenced fragments. This idea was tested using the simulation procedure implemented in rlsim software. Three different fragment lengths were considered: 250–450 (mean 350) bp, 450–650 (mean 550) bp, and 650–850 (mean 750) bp, with a fixed total read number of 1 million. The results are summarized in Figure 1E, with LOESS smoothing. It is difficult to give a precise position of the break point between the two regression lines, but it is clear that the greater the sequenced fragments, the more the break point is shifted toward the right. Moreover, the slopes for the regression lines situated before the break points seem to be similar.

To assess the efficiency of our method, we calculated the Pearson correlations between qRT-PCR and RNA-Seq counts corrected by FPKM (*Fragments Per Kilobase per Million mapped reads*) [23] or SGTR for the five bovine samples. We choose FPKM as it is a one of the most frequently method used for normalization. Briefly, it consists in dividing the fragment counts by transcript size and the total number of reads, and adjusted to 1 kb and 1 million reads.

**Figure 2:**
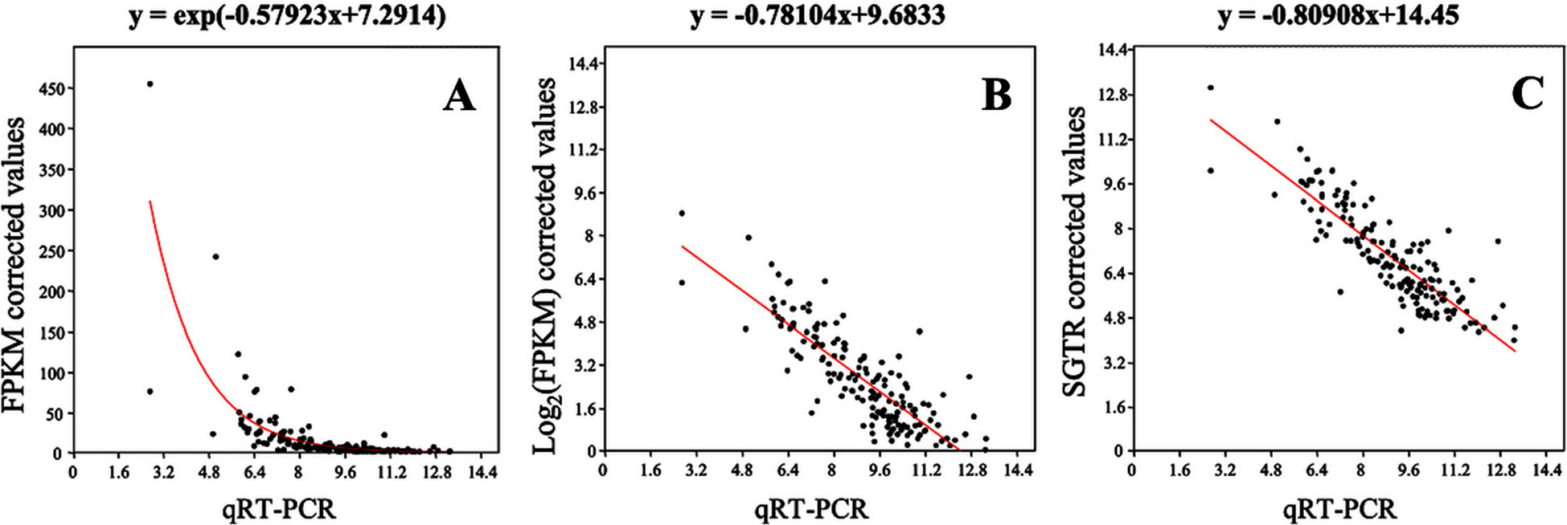
**Relationships between RNA-Seq normalization methods and qRT-PCR quantifications (Cattle sample 1479). A)** FPKM corrected values. **B)***log*_*2 *_(FPKM) corrected values. **C)** SGTR corrected values including size and GC content bias correction.

Among all the genes detected by qRT-PCR and RNA-Seq methods, we considered five sub-samples according to the class size of transcripts. The results shown in Table 1 indicate that the correction by FPKM is improved by transforming the raw values by their logarithms. Whatever the samples, the *p*-values observed for FPKM were largely worse than the one corresponding to *log*_*2*_(FPKM), except for transcripts < 1,000 bp and for transcripts ≥ 4,000 bp of the sample 1475 and 1455 which presented the lowest sequencing depth. This resulted from the distribution of values illustrated in Figure 2A and 2B. Consequently, further comparisons will only be made on the *log*_*2*_(FPKM) values. When we compare SGTR correction according to size only with the previous normalization, the *p*-values were generally of the same order of magnitude. Nevertheless, we observed slightly better results with our method for transcripts < 1,000 bp but faintly worse results for transcripts between 1,000 and 2,000 bp, whatever the sample.

**Table 1:** Comparison between FPKM and SGTR methods according to transcript size

	**N**	**Samples**	**FPKM**	***log***_***2***_**(FPKM)**	**SGTR - Size**	**SGTR - Size and GC content**
All genes	159	1475	1.07E-15	7.96E-39	1.71E-39	4.80E-39
Size < 1,000 bp	9	1475	9.59E-03	2.15E-02	1.58E-02	2.62E-02
1,000 – 2,000 bp	63	1475	1.86E-06	6.39E-18	4.71E-17	2.56E-17
2,000 – 3,000 bp	49	1475	2.81E-07	3.55E-14	1.75E-14	2.47E-14
3,000 – 4,000 bp	28	1475	3.25E-04	1.86E-06	1.86E-06	3.01E-06
Size > 4,000 bp	10	1475	1.56E-03	5.82E-03	6.95E-03	5.42E-03
All genes	155	1455	8.84E-16	2.95E-39	5.86E-39	3.40E-39
Size < 1,000 bp	9	1455	3.84E-02	1.03E-01	8.34E-02	1.05E-01
1,000 – 2,000 bp	60	1455	9.81E-07	1.57E-18	2.00E-17	4.19E-18
2,000 – 3,000 bp	50	1455	3.48E-09	9.07E-16	8.60E-16	1.43E-15
3,000 – 4,000 bp	26	1455	5.60E-05	2.80E-07	2.99E-07	4.50E-07
Size > 4,000 bp	10	1455	4.44E-03	2.26E-03	2.99E-03	1.96E-03
All genes	162	1479	4.63E-14	8.24E-44	1.37E-43	2.02E-49
Size < 1,000 bp	9	1479	7.54E-02	1.55E-01	1.18E-01	9.34E-02
1,000 – 2,000 bp	62	1479	1.75E-08	1.37E-16	6.87E-16	5.11E-18
2,000 – 3,000 bp	53	1479	1.68E-05	1.64E-19	1.64E-19	9.64E-22
3,000 – 4,000 bp	29	1479	1.67E-05	2.80E-09	3.08E-09	4.36E-10
Size > 4,000 bp	9	1479	1.33E-03	2.81E-05	5.39E-05	1.04E-04
All genes	152	1345	1.16E-14	1.57E-42	1.86E-42	6.11E-44
Size < 1,000 bp	9	1345	3.83E-02	9.84E-02	6.95E-02	7.98E-02
1,000 – 2,000 bp	58	1345	7.93E-08	6.39E-18	6.85E-17	2.43E-18
2,000 – 3,000 bp	50	1345	2.04E-05	8.39E-18	5.17E-18	1.74E-18
3,000 – 4,000 bp	26	1345	5.08E-03	7.67E-07	7.87E-07	1.01E-06
Size > 4,000 bp	9	1345	6.83E-04	1.62E-04	2.59E-04	4.44E-04
All genes	162	1476	2.73E-15	1.73E-41	6.74E-41	1.51E-44
Size < 1,000 bp	9	1476	5.12E-02	6.26E-02	4.52E-02	5.10E-02
1,000 – 2,000 bp	62	1476	3.38E-08	7.54E-17	4.72E-16	4.92E-18
2,000 – 3,000 bp	53	1476	1.55E-05	2.56E-17	3.53E-17	1.89E-18
3,000 – 4,000 bp	29	1476	3.44E-04	6.14E-07	7.13E-07	3.47E-07
Size > 4,000 bp	9	1476	9.18E-04	1.44E-04	2.76E-04	5.51E-04

N corresponds to the number of analyzed genes. The five samples (1475, 1455, 1479, 1345, and 1476) refer respectively to samples with a total read number around 10.10^6^, 13.10^6^, 20.10^6^, 24.10^6^, and 30.10^6^ reads. Abbreviations: SGTR size: correction for transcript size; and SGTR Size and GC content: correction for transcript size and GC content. Only the *p*-values of Pearson correlation with qRT-PCR quantifications are indicated. *p*-values were calculated using the Past3 program [24].

### Removing of the GC-content effect

**Figure 3:**
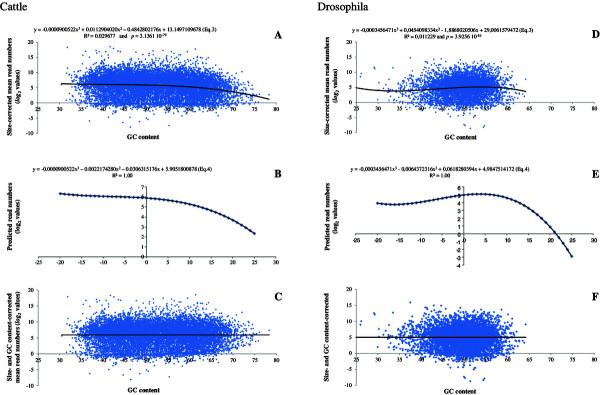
**Method implemented to correct GC content biases.** Variations in size-corrected mean read numbers according to GC content. The polynomial equations are indicated above (**A**: Cattle sample 1479, and **D**: Drosophila sample SRR384925). Application of the previous equation (Eq.3) to differences between 50% GC content and each GC content value, giving the equation indicated above (Eq.4) (**B**: Cattle sample 1479, and **E**: Drosophila sample SRR384925). Effect of GC content bias correction on the whole dataset. Clearly, no remaining dependence can be observed: the *p*-value to third order polynomial equation is 1.00 (**C**: Cattle sample 1479, and **F**: Drosophila sample SRR384925).

For the gene dataset of each cattle sample, we first calculated the trend curve for read numbers according to GC content. Polynomial equations of different order were tested and revealed dissymmetric dome shaped curves: the left increasing part (GC from 35 to 40%) was hardly visible by comparison to the right decreasing one (GC from 45 to 80%), where decreasing trend was getting more and more pronounced for GC > 50% (data not shown). We retained a third order polynomial function that clearly showed this last trend in all the samples, giving the Equation 3 (Figure 3A). Below the 50% threshold, the mean read numbers remained fairly constant.

(3) $$y=c.x^{3}d.x^{2}+e.x+f,$$

where ***y*** represents the size-corrected mean read number and ***x*** the GC content.Second, for each GC percentage, we calculated the difference between the GC content and 50% that we applied to the previous polynomial equation leading to the Figure 3B. The best fitting polynomial function was then deduced:

(4) $$y=g.x^{3}+h.x^{2}+i.x+j,$$

where ***y*** corresponds to the predicted read number and ***x*** represents the difference between a GC content and 50%.

Third, we adjusted the size-corrected values by removing “***g.x***^***3***^ ***+ h.x***^***2***^ ***+ i.x*** ” of this last function to all the transcripts. The Figure 3C illustrates the efficiency of GC bias correction.For the 20 human samples, we obtained the same profiles of size-corrected read number according to GC content as in Cattle (data not shown). In contrast, for the 16 drosophila samples, the polynomial curves were different (Figure 3D and 3E). Nevertheless, the correction of the GC content bias using the previous procedure yielded a smoothing curve absolutely flat (Figure 3F), attesting the efficiency of our method.

The final step consisted in testing the effect of the GC content bias removing on the correlation between RNA-Seq counting and qRT-PCR quantification, in the case of bovine data. Except for the sample 1475 (10.10^6^ reads), this last bias correction improved the global correlation with qRT-PCR quantifications relatively to the simple size correction by SGTR (Table 1). By comparison with *log*_*2*_(FPKM) correction, the removing of size and GC content biases improved the global correlation with qRT-PCR results, except for the sample 1455 which presented a low sequencing depth (13.10^6^ reads) and showed similar results as *log*_*2*_(FPKM) correction. The Figure 2C illustrates the correlation obtained between SGTR including size and GC content corrections and qRT-PCR quantifications for the sample 1479. We observed a better proportionality than the one provided by *log*_*2*_(FPKM) correction (Figure 2B).

### Adjustment according the total read number

**Figure 4:**
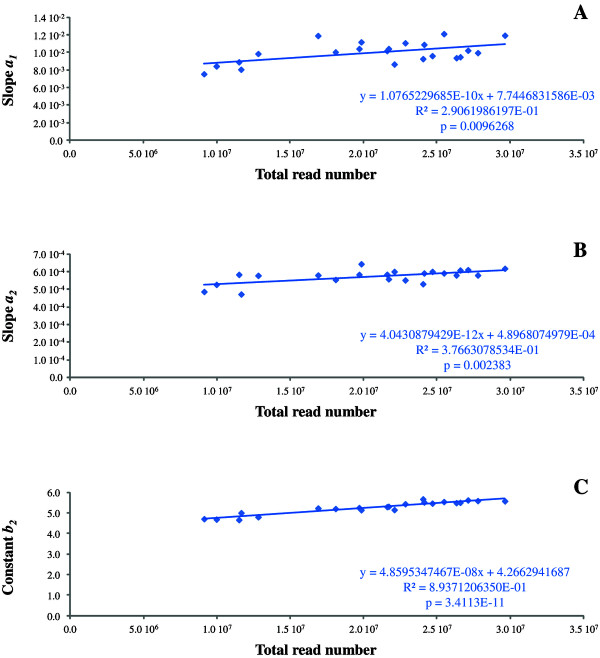
**Correlation between regression parameters and total read numbers (Cattle sample 1479). A)** Slope ***a***_***1***_ for transcripts < 600 bp. **B)** Slope ***a***_***2***_ for transcripts ≥ 600 bp. **C)** Constant ***b***_***2***_ for transcripts ≥ 600 bp. The equations are indicated below the regression lines.

For the 22 cattle samples, we calculated the correlations between total read numbers and the regression parameters (***a***_***1***_, ***b***_***1***_, ***a***_***2***_, and ***b***_***2***_) as given in Figure 1. Except the coefficient ***b***_***1***_, all the coefficients were positively correlated with total read numbers (Figure 4A, 4B, and 4C). The new regression parameters were defined as ***u***_***i***_ and ***v***_***i***_ for the slope and constant respectively:

(5) $$\;y_{i}=u_{1}.x_{i}+v_{1},$$

(6) $$y_{i}=u_{2}.x_{i}+v_{2},$$

and

(7) $$y_{i}=u_{3}.x_{i}+v_{3}$$

where ***y***_***i***_ corresponds to the regression parameter for a total read number ***x***_***i***_.

As 20 million was close to the mean value of total read numbers among the 22 samples, we decided to adjust all TRN (Total Read Numbers) to 20 million. For transcripts < 600 bp and TRN, we corrected the *log*_*2*_-transformed read numbers by adding the value “ ***u***_***1***_**(20.10**^**6**^**-*TRN*)** ” to the parameter ***a***_***1***_ in the Equation 1. Consequently, for a transcript of size ***S***, we corrected the values with the following equation:

(8) $$\begin{array}{l} y_{i}=\mathrm{lo}g_{2}\left(\mathrm{read}\;\mathrm{numbers}\right)\\ \;+\left[a_{1}+u_{1}\left(20.10^{6}-\mathrm{TRN}\right)\right]\left(600-S\right) \end{array}$$

where ***y***_***i***_ corresponds to the corrected read number for the transcript ***x***_***i***_.

Likewise, for transcripts ≥ 600 bp, we added the value “ ***u***_***2***_**(20.10**^**6**^**-*TRN*)** ” to the parameter ***a***_***2***_ in the Equation 2, and adjusted the corrected value by adding “ **[*u***_***3***_**( 20.10**^**6**^**-*TRN*)]** ”. As a result, the *log*_*2*_-transformed read numbers were corrected with the subsequent equation:

(9) $$\begin{array}{l} y_{i}=\mathrm{lo}g_{2}\left(\mathrm{read}\;\mathrm{numbers}\right)-\left[a_{2}+u_{2}\left(20.10^{6}-\mathrm{TRN}\right)\right]\\ \;\times \left(S-600\right)+u_{3}\left(20.10^{6}-\mathrm{TRN}\right) \end{array}$$

where ***y***_***i***_ represents the corrected read number for the transcript ***x***_***i***_.

On the other hand, after calculating the Eq.4’ corresponding to the Eq.4 based on the size- and TRN-corrected values, we determined the correlations between TRN and the regression parameters for GC content (defined as ***g’***, ***h’***, ***i’***, and ***j’***, as in Figure 3B). It appears that none of these parameters were linked to sequencing depth. Consequently, we corrected the GC content bias by removing “ ***g’.x***^***3***^ ***+ h’.x***^***2***^ ***+ i'.x*** ” to the size- and TRN-corrected values, giving the following equation:

(10) $$\begin{array}{l} y_{i}=\mathrm{lo}g_{2}\left(\mathrm{size}-\mathrm{and}\;\mathrm{TRN}-\mathrm{corrected}\;\mathrm{values}\right)\\ \;-\left(g’.x^{3}+h’.x^{2}+i’.x\right), \end{array}$$

where ***y***_***i***_ corresponds to the full-corrected read number, and ***x*** to the difference between the GC content and 50%.

Lastly, the negative final values were considered as null. It should be noted that when we applied the correction due to TRN, the correlations between SGTR and qRT-PCR quantifications became slightly better comparatively to the previous SGTR steps (Size and GC content corrections), except for the samples 1475 and 1476 which present the lowest and the highest sequencing depth (Table 2). In summary, the full SGTR correction showed better results than *log*_*2*_(FPKM), except for the sample 1475.

**Table 2:** Correction of the impact of total read numbers

	**N**	**Samples**	**log2(FPKM)**	**SGTR - Size**	**SGTR - Size and GC content**	**Full SGTR**
All genes	159	1475	7,96E-39	1.71E-39	4.80E-39	1.08E-38
	155	1455	2,95E-39	5.86E-39	3.40E-39	2.66E-39
	162	1479	8,24E-44	1.37E-43	2.02E-49	1.21E-49
	152	1345	1,57E-42	1.86E-42	6.11E-44	5.64E-44
	162	1476	1,73E-41	6.74E-41	1.51E-44	2.28E-44

N corresponds to the number of analyzed genes. The five samples (1475, 1455, 1479, 1345, and 1476) refer respectively to samples with a total read number around 10.10^6^, 13.10^6^, 20.10^6^, 24.10^6^, and 30.10^6^ reads. Abbreviations: SGTR size: correction for transcript size; SGTR Size and GC content: correction for transcript size and GC content; and Full SGTR: correction for transcripts size, total read number, and GC content. Only the *p*-values of Pearson correlation with qRT-PCR quantifications are indicated.

## Discussion

Our results showed that non-transformed counts values from RNA-Seq presented worse correlations with qRT-PCR quantification than the *log*_*2*_-transformed ones, as already stressed by [2,10,21], and [22]. The prior transformation of read counts by *log*_*2*_ function was motivated by the variability of data corresponding to highly expressed genes, often observed in large size transcripts. We hypothesized that this transformation could also attenuate the overestimations due to PCR duplicates. Indeed, the more expressed the transcripts, the higher the probability to generate duplicates (several clusters of reads share exactly the same start and end) [13,15]. Otherwise, certain authors have proposed to apply *log*_*2*_-transformed values to the data extracted from qRT-PCR [25,26]. Given our regression curves, it is clear that for our samples, this correction is inappropriate (unpublished data).

As for transcript size correction, two strategies have been adopted by different authors. In the first one, the transcripts are ranked in quantiles containing identical numbers [2,6,7]. The advantage is a balanced distribution facilitating further statistical analysis. However, it is difficult to assign a mean read number to scaled sizes. In the second one, size classes are built irrespective of the number of genes per class [4], leading to an increasing dispersion for the classes of higher sizes (mainly due to lower number of genes). Both approaches allowed avoiding certain limitations implemented in RPKM (*Reads Per Kilobase of exon model per Million mapped reads*) [1] or FPKM [23] methods, where the number of read is simply divided by transcript size. The main difference consists in taking into consideration paired-reads in the FPKM method while only simple reads in the RPKM one.

We choose the second strategy because of the excellent regression quality of mean read numbers by size classes. We interpret the border 600 bp observed whatever the species dataset (Figure 1A-1C) as a result of sonication and selection of cDNA fragments between 250 and 450 bp. Indeed, fragments > 600 bp are all the more so represented that they are long [1,3,4,27]. Conversely, the fragments < 600 bp are under-represented as many small segments were not sequenced. Moreover, the simulation conducted with rlsim confirmed our view, and showed that the border increases with the size of the sequenced fragments (Figure 1E). Hence, this proves the effect of the cDNA fragments size selection on the break point between the two regression lines. As a result, independent corrections are needed for both transcript sizes. This last point provided slightly better correction than the *log*_*2*_(FPKM) for transcripts < 1,000 bp (see Table 1). According to [14,28] and [29], RNA-Seq protocol including PCR in the first steps introduced biases linked to GC content, as cDNA fragments with high GC and AT content are under-sequenced. To correct this bias, [10,14] and [30] proposed to build GC-classes. In our method, we took into account the general trend by calculating a three order polynomial equation, which was used to correct the decrease over 50% GC content. The efficiency of our correction was sample-dependent and more precisely linked to sequencing depth. Indeed, for a low number of reads, the GC bias correction did not improve the normalization, in contrast to samples with higher sequencing depth. SGTR including Size and GC content corrections provide thus globally better results than *log*_*2*_(FPKM) (Table 2), which is in agreement with the conclusions of [8] and [10]. We expect that the GC content correction should be more accurate if it was applied on gene segments (~300 to 500 bp) and not on full length transcripts, as there are variations along the sequence in their GC content.

Lastly, since the sequencing depth introduces effects on transcript size bias, we adjusted the TRN to 20 million reads in reason of its medium value. Hence, we modified the parameters ***a***_***1***_, ***a***_***2***_, and ***b***_***2***_, but this step requires numerous samples to obtain reliable values. Finally, these size and TRN adjusted values were then corrected for GC content bias.

Our integrated method corrects some biases linked to transcript size and GC content, but also sequencing depth. However, it is striking that for the lowest sequencing depths (sample 1475: 10 million reads; 1455: 13 million reads) our correction gave worse or equal correlations with qRT-PCR values than *log2*(FPKM). In contrast, for read counts over 20 million, our method significantly improves the read counting, for the whole dataset and for most gene size classes. The question is to interpret this observation and several considerations have to be taken into account. First, in our samples, when the total number of reads is low, it is particularly true for transcript with sizes shorter than 600 pb, the regression equation between transcript size and read counts is less accurate than the one for transcript sizes longer than 600 pb. Second, the more expressed the transcripts (total read numbers over 20 million), the higher is the probability to generate duplicates and other biases induced by RNAseq. Our method can be compared to GAM (*Generalized Additive Model*) of [11], where the data are corrected for length, GC content, and dinucleotide frequency biases. However, these authors have shown that the correction of dinucleotide frequency biases did not improve results. Unlike GAM method, our model is not additive as we showed that the regression coefficient linked to transcript length depend on the sequencing depth. That was not the case for polynomial equation coefficients used to correct the GC content bias. Improvements are still needed to better take into account the variation of GC content per read in a given transcript, as the GC content is not homogeneous along the sequence. Protocols excluding PCR in first step could avoid this issue, and problems linked to PCR duplicates [13,15,28]. On the other hand, it is highly desirable to provide a good estimation of the number of reads corresponding to each transcript isoform. To overcome this issue, we took into account genes presenting only one transcript. In contrast to Human [11], this choice does not result in a dramatic loss of information as more than 50% of bovine genes have a single transcript in the available annotation file. The accurate determination of transcript size suffers from biases linked to cDNA library preparation. Indeed, it seems that random-hexamers present some favored and disfavored sites, so that specific regions are selected more easily than others leading to biases for low expressed genes [1,31,32]. RNA fragmentation before its reverse-transcription in cDNA reduces this bias leading to more uniform gene coverage [33]. Nevertheless, these technical effects associated to library preparation as well as some variations observed between flow cells have always a smaller influence that the biological effect [6,9]. Otherwise, the fine determination of TSSs (Transcription Start Sites) deduced from alignment of the reads onto the genome (and not onto the known transcripts) could further improve the accuracy of transcript size.

## Conclusions

We demonstrated that our method is robust and suitable to compare the read counts of genes for numerous samples of the same tissue. All the steps described are sequentially automated within SGTR program written in Perl, and available upon request from RP and DP. The extension of our method to the normalization of the read numbers between different tissues requires considering a set of reference genes as calibrators.

### Animal ethics

All animal experimentation complied with the French Veterinary Authorities’ rules. No ethics approval was required by a specific committee, as the selected animals were not animals bred for experimental reasons.

## Competing interests

The authors declare that they have no competing interests.

## Authors’ contributions

AM and DP conceived the study. CM prepared the RNA samples and performed the RNA-Seq experiments. CF performed the qRT-PCR analysis, under the supervision of LF. CF and CM analyzed the data and developed the method. CK processed the RNA-Seq data from bovine samples and from public datasets. RP implemented the software. CM and DP drafted the manuscript. AM, DP and DR obtained the different funds. DR was the initiator of the bovine RNAseq project. All authors read and approved the final manuscript.

## Supplementary Material

Additional file 1: Table S1 Absence of significant correlations between qRT-PCR data and transcript sizes or GC contents. N corresponds to the number of analyzed genes. The five samples (1475, 1455, 1479, 1345, and 1476) refer respectively to samples with a total read number around 10.10^6^, 13.10^6^, 20.10^6^, 24.10^6^, and 30.10^6^ reads. We indicated the *p*-values associated to polynomial (first and third orders) regression equations between ΔCT values and transcript sizes or GC contents. Click here for file
