# Supplementary material for: An integrative method to normalize RNA-Seq data
Source: BMC Bioinformatics. 2014 Jun 14;15:188. doi: 10.1186/1471-2105-15-188 (PMC4067528; doi:10.1186/1471-2105-15-188)
Supplement: Additional file 1: Table S1 — Absence of significant correlations between qRT-PCR data and transcript sizes or GC contents. N corresponds to the number of analyzed genes. The five samples (1475, 1455, 1479, 1345, and 1476) refer respectively to samples with a total read number around 10.106, 13.106, 20.106, 24.106, and 30.106 reads. We indicated the p-values associated to polynomial (first and third orders) regression equations between ΔCT values and transcript sizes or GC contents. [file 1471-2105-15-188-S1.docx]

**Additional file 1: Table S1: Absence of significant correlations between qRT-PCR data and transcript sizes or GC contents.**

N corresponds to the number of analyzed genes. The five samples (1475, 1455, 1479, 1345, and 1476) refer respectively to samples with a total read number around 10.10^6^, 13.10^6^, 20.10^6^, 24.10^6^, and 30.10^6^ reads. We indicated the *p*-values associated to polynomial (first and third orders) regression equations between ΔCT values and transcript sizes or GC contents.

|  |  | **Transcript size** | | **GC content** | |
| --- | --- | --- | --- | --- | --- |
| **Sample** | **N** | **First order** | **Third order** | **First order** | **Third order** |
| 1475 | 227 | 0.56934 | 0.82165 | 0.31301 | 0.39519 |
| 1455 | 227 | 0.75083 | 0.87375 | 0.44471 | 0.63522 |
| 1479 | 233 | 0.83638 | 0.86532 | 0.57027 | 0.20691 |
| 1345 | 231 | 0.76718 | 0.89059 | 0.94616 | 0.17653 |
| 1476 | 233 | 0.58959 | 0.81628 | 0.34675 | 0.23948 |
